# New Approaches and Technologies to Improve Accuracy of Acute Otitis Media Diagnosis

**DOI:** 10.3390/diagnostics11122392

**Published:** 2021-12-19

**Authors:** Susanna Esposito, Sonia Bianchini, Alberto Argentiero, Riccardo Gobbi, Claudio Vicini, Nicola Principi

**Affiliations:** 1 Pediatric Clinic, Pietro Barilla Children’s Hospital, Department of Medicine and Surgery, University of Parma, Via Gramsci 14, 43126 Parma, Italy; Bianchini.sonia@outlook.it (S.B.); alberto.argentiero@unipr.it (A.A.); 2 Head-Neck and Oral Surgery Unit, Department of Head-Neck Surgery, Otolaryngology, Morgagni Piertoni Hospital, 47121 Forlì, Italy; riccardo.gobbi@auslromagna.it (R.G.); claudio@claudiovicini.com (C.V.); 3 Università degli Studi di Milano, 20122 Milan, Italy; nicola.principi@unimi.it

**Keywords:** acute otitis media, artificial intelligence, smartphone otoscopy, telemedical otoscopic examination, video-otoscopy

## Abstract

Several studies have shown that in recent years incidence of acute otitis media (AOM) has declined worldwide. However, related medical, social, and economic problems for patients, their families, and society remain very high. Better knowledge of potential risk factors for AOM development and more effective preventive interventions, particularly in AOM-prone children, can further reduce disease incidence. However, a more accurate AOM diagnosis seems essential to achieve this goal. Diagnostic uncertainty is common, and to avoid risks related to a disease caused mainly by bacteria, several children without AOM are treated with antibiotics and followed as true AOM cases. The main objective of this manuscript is to discuss the most common difficulties that presently limit accurate AOM diagnosis and the new approaches and technologies that have been proposed to improve disease detection. We showed that misdiagnosis can be dangerous or lead to relevant therapeutic mistakes. The need to improve AOM diagnosis has allowed the identification of a long list of technologies to visualize and evaluate the tympanic membrane and to assess middle-ear effusion. Most of the new instruments, including light field otoscopy, optical coherence tomography, low-coherence interferometry, and Raman spectroscopy, are far from being introduced in clinical practice. Video-otoscopy can be effective, especially when it is used in association with telemedicine, parents’ cooperation, and artificial intelligence. Introduction of otologic telemedicine and use of artificial intelligence among pediatricians and ENT specialists must be strongly promoted in order to reduce mistakes in AOM diagnosis.

## 1. Background

Acute otitis media (AOM) is mainly a bacterial disease, although an acute viral upper-respiratory tract infection, including that due to SARS-CoV-2 [1], often precedes the development of the signs and symptoms of disease in most cases [2]. This explains why, when strong containment measures on the circulation of respiratory viruses are implemented, as recently occurred during the coronavirus disease 2019 (COVID-19) pandemic, the incidence rate of AOM reduces [3]. *Streptococcus pneumoniae*, nontypeable *Haemophilus influenzae, Moraxella catarrhalis*, and *Streptococcus pyogenes* are the most common bacterial pathogens that cause AOM [2]. Several studies have shown that in recent years, even outside the pandemic period, incidence of AOM has declined worldwide [4,5,6]. In the USA, between 2011 and 2016 the annual incidence of AOM in children aged 0–9 years was reduced by about 25% [7]. Advances in antibiotic treatment strategies, widespread adoption of potentially effective preventive measures, and greater adherence to scientific society guidelines may have contributed to the achievement of these reductions [8]. However, incidence of AOM and its related medical, social, and economic problems for patients, their families, and society remain very high. By 3 years of age, 60% of children have suffered ≥1 episode and 24% ≥3 episodes, making AOM the most common cause of antibiotic use in the pediatric age-group [9].

Better knowledge of potential risk factors for AOM development and more effective preventive interventions, particularly in AOM-prone children, can further reduce disease incidence. However, a more accurate AOM diagnosis seems essential to achieving this goal. Diagnosis of AOM using clinical criteria and standard instruments remains challenging [2]. Diagnostic uncertainty is common, and to avoid risks related to a disease mainly caused by bacteria, several children without AOM are treated with antibiotics and followed as true AOM cases [2]. Excessive antibiotic prescription, increased prevalence of antimicrobial resistance, unnecessary tympanostomy tube procedures, excess days off school for children and work for parents, and relevant reduction in quality of life for all the family are the most important consequences of misdiagnosis [5,10,11,12,13,14].

To face all the mentioned problems, a different approach to AOM diagnosis has been advocated by several scientific societies. Artificial intelligence has been used to train computer software in otoscopic images of the TM improving diagnostic accuracy by pediatricians and extending child evaluation to parents. New devices that are more effective in defining tympanic-membrane (TM) modifications and evidence of middle-ear effusion (MEE) have been developed [15,16,17]. The main objective of this manuscript is to discuss the most common difficulties that presently limit accurate AOM diagnosis and the new approaches and technologies that have been proposed to improve disease detection.

## 2. Currently Used Methods to Diagnose Acute Otitis Media (AOM)

**Figure 1:**
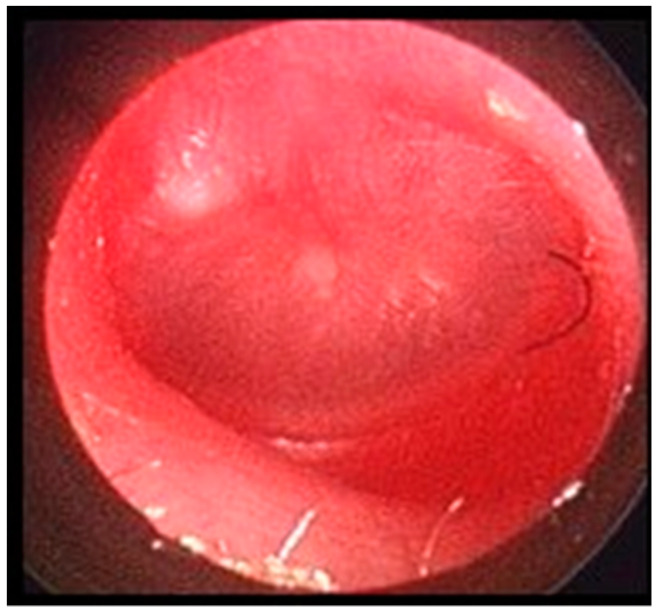
Tympanic membrane with intense erythema and bulging in a 2-year-old child with ear pain and fever.

Official national guidelines uniformly indicate that AOM diagnosis can be made only when the following criteria are simultaneously present: (1) acute onset of otalgia or, in preverbal children, symptoms suggesting ear pain, such as tugging, rubbing, or holding the ear, with or without fever; (2) signs of TM inflammation, featuring as intense erythema or yellow color of the eardrum; and (3) presence of MEE, which can appear as bulging of the TM or otorrhoea, or is strongly supposed on the basis of greatly reduced/absence of mobility of the eardrum [14,17,18,19]. Figure 1 shows the TM of a 2-year-old child with a confirmed diagnosis of AOM.

Neither systemic nor ear-specific symptoms are sensitive or specific enough in the diagnosis of AOM. Otalgia can be lacking in 35% of older children and in 50% of those <2 years [20]. Infant’s behavior may be incorrectly interpreted by parents [21,22]. Fever is reported only in about 50% of the AOM episodes [23,24].

**Figure 2:**
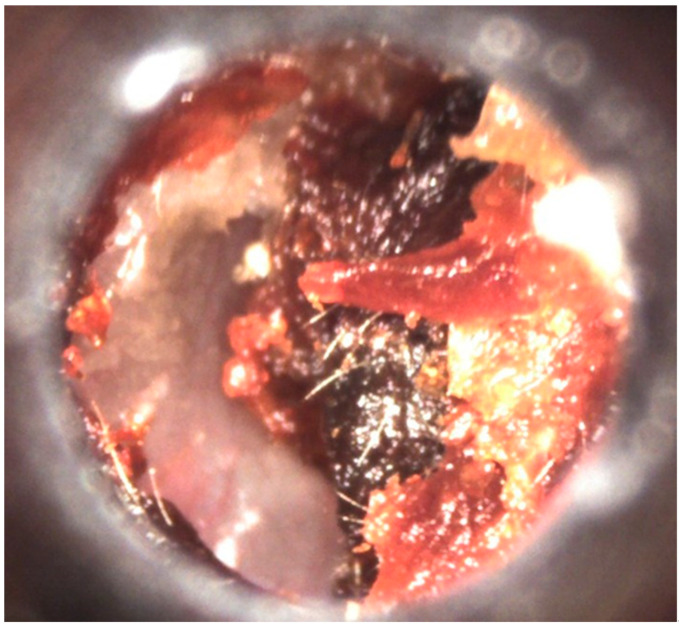
Ear canal partially blocked by cerumen in a 3-year-old child.

Evaluation of TM inflammation and presence of MME is not easy and, even when possible, does not always allows a correct diagnosis. Problems include the characteristics of the current instrument used to visualize the TM, the physician’s ability, and the sensitivity and specificity of ear findings. In emergency rooms and in office pediatric and general practitioner practice, to evaluate TM inflammation, bulging or perforation, a standard otoscope is generally used. This is a monocular device that provides only a two-dimensional view of the ear canal and does not allow evaluation of eardrum mobility or definition of AOM etiology. Moreover, the visualization of MT can be difficult or incomplete for at least two reasons. Lightning can be inadequate as physicians can pay poor attention to battery charge or bulb efficiency. A study has shown that approximately 30% of physicians exchange the otoscope bulbs less often than recommended, and 30% of otoscopes do not have adequate lighting capacity [25]. In addition, the ear canal can be partially or totally blocked by cerumen (Figure 2).

Visualization of the whole TM, including light reflex, ossicles, and mobility, requires that more than 75% of the ear canal diameter must be free [26]. More than 70% of children have cerumen and, among these, more than 40% have an ear canal obstruction ≥50%. Unfortunately, the attitude of pediatricians towards cerumen removal seems very poor, frequently leading to diagnosis based on criteria different from those recommended as cerumen was not adequately removed [27].

However, even when TM characteristics can be adequately evaluated, results can leave doubts. TM findings are not sufficiently specific and sensitive for AOM diagnosis. Intense erythema or yellow color of the TM are not detected in about 20% of children with true AOM. On the contrary, a slightly red TM is common in children with upper respiratory tract infection without true ear involvement [28]. Moreover, in a crying child the TM can be mistaken for AOM because of the presence of erythema or dilation of TM vessels [29]. Compared to tympanocentesis, membrane bulging suggested by means of standard otoscopy has high specificity (up to 97%) but poor sensitivity (51%).

To improve efficiency of standard otoscopic examination, otomicroscopy was introduced. It shows an enlarged view and binocular viewing, which permits depth perception, complete evaluation of the TM and, when needed, an easier removal of cerumen. In detection of MEE, otomicroscopy was found superior to standard otoscopy as sensitivity and specificity were 87–91% and 89–93%, respectively [30,31]. However, the otomicroscope has some limitations which, although easily overcome by otolaryngologists especially in the surgical setting, make it difficult for pediatricians and general practitioners to use it in their daily outpatient practice. It is a very expensive machine, has important space limitations, does not permit an etiological diagnosis, and to be used without significant misdiagnoses requires accurate training.

**Figure 3:**
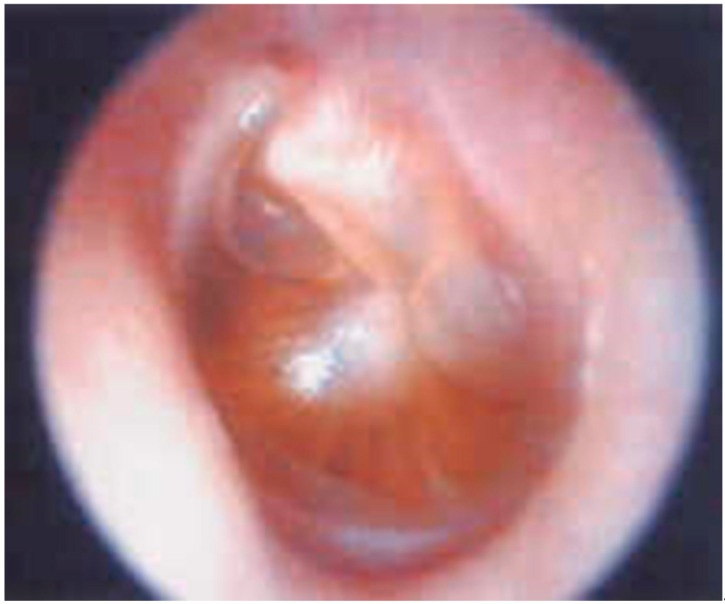
Tympanic membrane with middle-ear effusion but without signs of acute inflammation in a 1-year-old child.

The reference method to detect MEE is tympanocentesis, that allows not only the evaluation of the presence of MEE but also its characteristics, differentiating true AOM from otitis media with effusion (OME). Figure 3 shows the TM of a 1-year-old child with a diagnosis of OME.

Moreover, it assures culture of MEE and susceptibility testing of isolates so allowing adequate antibiotic treatment. This explains why tympanocentesis has been recommended to guide the choice of antibiotics in difficult AOM episodes [32,33] and the Centers for Disease Control and Prevention of the USA have informed that clinicians should consider developing the capacity to perform tympanocentesis [33]. Unfortunately, tympanocentisis is an invasive method that cannot be used in the primary-care setting. It requires skill and training, involves the presence of a nurse, is a time-consuming procedure, and is not viewed with high favor by parents. CIt remains an option only for studies comparing different methods to diagnose AOM, for clinical trials evaluating antibiotic efficacy, and for children requiring precise identification of AOM etiology, i.e., those with recurrent episodes, or at high risk for severe outcome, such as neonates or immunocompromised patients. On the other hand, it can be performed only when a bulging TM is documented and remains a method that must accompany TM examination through otoscopy [34].

To overcome limitations of standard otoscopy in MEE detection, a series of instruments such as the pneumatic otoscope (PO), tympanometer (TP), and acoustic reflectometer (AR) have been developed over recent years. The PO allows evaluation of the TM optical characteristics exactly as standard otoscopy, but it is significantly more effective in the assessment of MEE. PO measures TM mobility through the deflection of the TM under pressure revealing presence of fluid behind the TM when the deflection is scarce or completely absent. Compared to standard otoscopy, PO has shown improvement in sensitivity and specificity for AOM diagnosis of 24% and 42%, respectively [35]. For these reasons, PO is indicated by most official national guidelines as the simplest and the most effective method to diagnose OMA in everyday practice, particularly in outpatient setting [14,17,18,19]. However, in routine office practice it has several problems that limit its use. PO is difficult to perform especially in younger children because of the narrow ear canal and tendency to wriggle [36]. Despite being more effective than standard otoscopy for MEE detection, PO does not allow identification of the type of MEE and, consequently, it does not distinguish AOM from OME and does not give information on the most appropriate antibiotic therapy, if needed. Moreover, it is a subjective evaluation that is based on the experience of the operator [37] and requires extensive training or extended practice to lead to satisfactory results [38,39]. A previous study showed that the sensitivity and specificity to diagnose MEE increased from 58% to 67% and from 78% to 81%, respectively, according to the level of physician experience [40]. All these findings explain why pediatricians and general practitioners use PO very little. A survey carried out among family-medicine residents reported an occasional use by 66% and consistent use by only 15% [41]. Among pediatricians, an American study showed that only 21% of physicians always used PO and 42% never used it [42]. A recent Italian evaluation confirmed these findings, showing that primary-care pediatricians routinely used PO only in 9.6% of the suspected AOM cases [43].

TP and AR can help physicians in the identification of MEE although they do not give any information on the TM visual status and, therefore, can only be used in association with otoscopy. Moreover, TP, as PO, does not allow evaluation of the type of fluid present behind the TM and does not differentiate AOM from OME. TP evaluates variations in acoustic impedance of the TM/middle-ear system with air pressure variations in the ear channel and has the advantage of not being influenced by cerumen unless it occupies more than half of the ear canal diameter [44]. Tympanometry has the disadvantage of requiring an airtight seal in the ear canal, and the accuracy of the results is thus affected by the cooperation of the child [45]. Tympanogram tracings are classified as type A (normal), type B (flat, clearly abnormal), and type C (showing a significantly negative pressure in the middle ear, possibly suggestive of pathology). Moreover, type C tracings can be further differentiated in C1, C2, and Cs according to the risk of MEE presence. As many children, even among asymptomatics, have a type C curve, this means that TR can be frequently useful only when associated with other findings, but by itself it is frequently an imprecise estimate of middle-ear pressure and does not have high sensitivity or specificity for middle-ear diseases. This explains why study results vary according to the type of instrument and the pressure of the air, and the tympanogram is considered abnormal. Using only type B tympanograms as abnormal, the TP was found to have sensitivity of 80.9% (95% confidence intervals (CI): 76.1–85.7) and specificity 74.5% (95% CI: 66.9–82.0). When both type B tympanograms and a C2 curve were considered together as abnormal findings, overall sensitivity rose to 93.8% (95% CI: 91.1–96.4), but specificity fell to 61.8% (95% CI: 41.5–82.1) [46]. Studies comparing TP and PO for MME detection have shown that the two methods have similar ability or that TP is slightly less effective [47,48]. Evaluation of the accuracy of methods of diagnosing MEE in pediatric patients revealed that among eight diagnostic methods used between 1980 and 2000, PO had the best performance with a sensitivity of 94% (95% CI: 92–96) and a specificity of 80% (95% CI: 75–86). Professional tympanometry had the highest specificity (94.1%; 95% CI: 83.9–100) but pneumatic otoscopy optimized both sensitivity and specificity [49]. Combined use of TP and PO can further improve AOM diagnosis A study revealed that in this case sensitivity and specificity was increased to more than 90% [50].

Similar results have been obtained with AR. This procedure evaluates the acoustic response of the TM to a sound emitted by an instrument. Practically, the degree to which the reflected sound waves cancel the incident waves emitted by the instrument is measured. The most advanced instruments calculate and display the data from incident and reflected sound graphically as a curve, from which the spectral gradient angle can be evaluated. If the movement of the TM is limited by fluid in the middle ear, the membrane reflects more sound energy, and the spectral gradient curve is different from that obtained in a normal subject. Different pattern categories representing different probabilities of MEF have been calculated [51]. Compared to PO and TP, AR has some practical advantages as it does not need an airtight fit in the ear canal and can also be successfully obtained in an uncooperative patient. Unfortunately, the size of the ear canal, the middle-ear pressure, the position of the TM, and the amount of MEE may all influence the spectral gradient curve making results debatable [52,53]. Finally, a supine position [54] and anesthesia [55] can significantly influence AR accuracy for MEE detection. Comparative studies have generally reported that the accuracy of AR for MEE detection is quite like that of PO and TP or, in some cases, PO and TP in validated hands are slightly more accurate [44,56,57,58].

## 3. Improvement of Traditionally Used Instruments for Acute Otitis Media (AOM) Diagnosis

The most important problem for a reliable AOM diagnosis in everyday practice of primary-care pediatricians and general practitioners is the evidence that whatever instruments is used, results of examination are strictly dependent on the physician’s experience and interpretations [59]. To increase the diagnostic accuracy of AOM, physicians should be up-to-date and medical students and residents appropriately educated. Unfortunately, this not always occurs. To overcome this problem, attempts to improve the quality of visual otoscopic images have been made, in some cases with important advances.

Video-otoscopes are the base for a more accurate TM evaluation even when otoscopy is not made by a validated physician. Compared to standard otoscopes, video-otoscopes have the advantage of allowing the collection of a great number of images of the TM that, contrary to what happens when standard otoscopy is performed, can be later reviewed by the physician himself or a specialist. This allows a deeper analysis of the TM characteristics and reduces the risk of misdiagnosis [60]. Moreover, images can be shown to the parents who can then more easily accept further instrumental tests and therapy if needed. A good example in this regard is given by the use of a smartphone otoscope attachment called CellScope Oto^®^ (CSO), consisting of a portable video-otoscope that permits sharing of diagnostic-quality video and images. Several studies have evaluated the accuracy of this and other similar instruments for the diagnosis of AOM [61,62,63,64,65]. Results indicate that they are at least as effective as a standard otoscope and, in some cases, can significantly improve diagnostic accuracy without adding advantages in etiological diagnosis. In a study in which video otoscopy was evaluated by an ear, nose, and throat (ENT) specialists, audiologists, and trained research assistants, more video otoscopy recordings were evaluated as ‘good’ or ‘excellent’ in comparison still images [65]. Moreover, video otoscopy can be used for teaching and can improve the ability of poorly experienced physicians. In a prospective cross-sectional study, children with suspected AOM were examined by residents and attending physicians using both a traditional otoscope and the CSO [55]. Intra-rater and inter-rater agreements were evaluated. Results showed that residents and attending physicians overwhelmingly agreed that CSO was easy to use, enabled more precise diagnosis, enhanced TM visualization, and was a good teaching tool. Final diagnosis was changed several times, with significant influence on antibiotic prescription rates [62]. Moreover, in a research carried out in adults, CSO was found 96% specific in the diagnosis of normal TM and 100% sensitive in the diagnosis of disease [66]. Overlapping results were obtained with a similar instrument, the Cupris^®^ smartphone device [67]. The rates of sensitivity, specificity, positive predictive value (PPV), and negative predictive value (NPV) of the Cupris^®^ device to diagnose any middle-ear disease were 94%, 96%, 91%, and 97%, respectively.

Further improvement was obtained when it was possible to compare images collected with video-otoscopes with a series of TM images previously collected in patients with well-defined ear diseases. The correspondence with what was highlighted in the patient under study with the characteristic findings of certain diagnoses allowed many doubtful cases to be resolved, increasing the number of correct diagnoses [68]. Feature-extractions-based algorithms for automatic diagnosis of ear diseases have been developed. A recent example in this regard has been reported by Livingstone and Chau [69]. A total of 1366 otoscopic images related to 14 well-defined otologic diagnoses were obtained from patients visited in a number of associated institutions and from Google Images (Google Inc., Mountain View, CA, USA). They were uploaded to the Google Cloud Vision AutoML platform (Google Inc.) and a multilabel classifier architecture algorithm was trained. Diagnostic performance of the algorithm was compared to the performance of physicians using the same test set of images [69]. For all diagnoses combined, the positive predictive value of the algorithm was 90.9%, and the sensitivity was 86.1%. Accuracy of the algorithm was 88.7%, whereas that of physician was 58.9%. A system strictly related to AOM and OME diagnosis has been studied by Wu et al. [70]. These authors developed a brief method for automated classification of otitis media diseases using conventional neural networks such as Xceptions and MobileNet-V2 together with images from otoscope. For all diagnoses combined, the two convolutional neural networks had similar accuracies of 97.4% (95% CI: 96.8–97.9) and 95.7% (95% CI: 95.1–96.1) [70]. Even more advanced analysis can be obtained with the HearScope system [71]. In this case, two artificial intelligence systems for classifying otoscopy images are used. The first screens whether the image is of the ear canal and the TM. If an ear canal is confirmed, the second neural network classifies the images into one of four categories—normal, wax obstruction, chronic perforations, or abnormal. The current system that has been released has a 94% accuracy for classifying images into the four currently supported diagnostic categories [71].

Another development in otoscopy involves telemedical otoscopic examination (TOE). Instruments such as smartphone otoscopes can be used by parents and images strongly suggesting ear disease can be sent to the primary-care pediatricians or an ENT specialist for confirmation. Particularly in AOM-prone children, with TOE examination a number of visits in emergency departments and on pediatric wards could be avoided, and documentation of captured images or videos for future reference could be obtained. Although this technique cannot permit an etiological differentiation, it could be used to reduce antibiotic abuse because of an inappropriate AOM diagnosis. Theoretically, TOE could be easily introduced in routine clinical practice as parents can easily learn to use the otoscope. Erkkola-Anttinen et al. reported that after a simple teaching intervention during a visit for a previous ear disease, healthy ear, OME, or AOM could be diagnosed in 40% of the videos collected by parents [72]. Moreover, in all the studies in which smartphone otoscopy examination was evaluated most of the parents were positive about instrument use. Almost all agreed that the images helped them to understand the child’s condition and the chosen management. About 90% felt comfortable about using it and following the middle-ear status at home over time. Two-thirds declared that they preferred sending images to their physician rather than making an office visit [62,65]. However, despite parents’ favorable opinion, use of TOE does not seem to be considered favorable by physicians. Despite the fact that in a survey it was reported that pediatricians agreed on the potential value of the TM images to educate families, and 66% considered that following the images over time could even decrease the administration of antibiotics for AOM therapy, most of them had doubts about losing the hands-on examination and the direct patient contact [65]. Overall, 38% agreed that they would have administered antibiotics based on a remote AOM diagnosis. Moreover, 63% estimated that parents could not perform or obtain a video examination of their child’s TM and that the images would be of poor quality due to cerumen and lack of testing the TM movement [65].

## 4. New Measures for Diagnosis of Acute Otitis Media (AOM)

Recently, more advanced technologies able to better define TM characteristics and MEE presence compared to the standard otoscope and other instruments currently used for AOM diagnosis, have been developed [16,73,74]. Unfortunately, these new technologies have not yet been used for the production of instruments that can be used in the outpatient practice of primary-care pediatricians and general practitioners. In some cases they are very interesting and can be considered promising options for improving AOM diagnosis. Among technologies with the most advanced development, are light-field otoscopy (LFO), optical coherence tomography (OCT), low-coherence interferometry (LCI), and Raman spectroscopy (RS).

Standard otoscopy and video otoscopes do not allow a 3-dimensional reconstruction of the TM. This may lead to the misinterpretation of the presence of bulging of the TM, contributing to the most common clinician error—classification of OME as AOM. To overcome this problem, LFO based on a regular camera lens associated with a light-field camera has been developed [75]. Multi-view images of the TM can be collected, a 4-dimensional structure called the light field is formed and, finally, a 3D picture can be computed. Reconstructions of the TM in normal, AOM, and OME have been acquired [75]. No information on its impact in etiological diagnosis is available.

OCT is a non-invasive technology that allows the reconstruction of a depth-resolved, high-resolution, cross-sectional image of the TM using a low-power, near-infrared light source. With a handled probe quite similar to a standard otoscope, total thickness of the TM and characteristics of any of its three layers can be measured and compared with those of a normal TM [76]. As during AOM the TM is significantly thickened, the intensity of the backscattered light is reduced, making the tissue characteristics less bright on the OCT image [77]. Moreover, MEE can be detected and differentiation of serous from purulent fluid can be achieved [78]. Efficacy of OCT in this regard has been recently evaluated in a study enrolling children undergoing tympanostomy-tube placement. Analysis of OCT data resulted in 90.6% accuracy, 90.9% sensitivity, and 90.2% specificity [78]. Differentiating MEE type, identification of nonserous MEE had 70.8% accuracy, 53.6% sensitivity, and 80.1% specificity. The age of patients was critical for OCT quality. The mean age of subjects with quality OCT was 5.01 years compared to 2.54 years in the remaining subjects (*p* = 0.0028). Moreover, the instrument used in this study had several practical limitations that require solution for a more easy and effective use [79].

LCI is an optical-ranging technique that can produce high-resolution, depth-resolved scans of the TM based on its optical reflectivity [80]. It can be considered an improvement on traditional PO. Contrarily to PO, whose accuracy relies on the physician’s experience and expertise, LCI provides a quantitative evaluation of the TM mobility trough objective and quantitative metrics (compliance and amplitude ratio). As pressure is computer-controlled, the problem of the variability of the pressure pulses between physicians is eliminated. Moreover, as the strength of the air puff can be varied and very low pressure can be used, even mild alterations of TM mobility not visible to the naked eye with the PO can be detected. Pneumatic LCI otoscopy can give more dynamic information for subjects with MEE than tympanometry, and directly measure TM displacements with potentially gentler and more precisely controlled air-pressure transients than PO [81]. No additional information on etiological diagnosis can be reached with this technique.

RS is an analytical technique where scattered light is used to evaluate the vibrational-energy modes of a sample [55]. It is generally able to identify chemical and structural information, as well as being able to identify substances through their characteristic fingerprint. Moreover, presence of bacteria and identification of species is possible [80]. In experimental animals, RS was found able to identify early inflammation changes of the TM, suggesting its use for AOM diagnosis in the first phase of disease [82]. Moreover, in children, differentiation of AOM from OME based on the spectral markers (i.e., mucin), with classification accuracy of 91% and 93% for serous and mucoid, respectively, was achieved [83].

## 5. Conclusions

AOM is a common disease that needs a prompt and accurate diagnosis. Misdiagnosis can be dangerous or lead to relevant therapeutic mistakes. In most of the cases, AOM is a bacterial disease and failure to diagnose it can lead to the development of extremely dangerous, sometimes fatal, complications (i.e., mastoiditis, meningitis, or cerebral abscess). A diagnosis of AOM in a subject who suffers from OME or who has no ear problems can lead to an exaggerated use of antibiotics or to an unnecessary follow-up. To achieve this goal an essential prerequisite is that the doctor who visits the child has had adequate training on AOM diagnosis and that he/she knows how to use rationally the tools that the technique has made available for this purpose

For several years, diagnosis of AOM was based on the use of otoscope, a very old instrument with several known limitations that make diagnosis difficult even in validated hands. Unfortunately, the introduction of the otomicroscope, PO, TP, and RF has only slightly improved standard otoscopy ability to diagnose AOM, mainly because their use and the correct interpretation of findings are difficult. The need to improve AOM diagnosis has led to the development of a long list of new methods to visualize and evaluate the TM and to assess MEE. Most of the new instruments, such as those based on the most modern technologies, are far from being introduced into clinical practice and presently remain interesting tools without real practicability in everyday pediatrician and general practitioner activity. More effective can be video otoscopy, especially when it is used in association with telemedicine, parents’ cooperation, and artificial intelligence. Every effort must be made to increase, as much as possible, the technical expertise of students and residents in using video-otoscopy. Moreover, introduction of otologic telemedicine and the use of artificial intelligence among pediatricians and ENT specialists must be strongly promoted in order to reduce mistakes in AOM diagnosis and reduce inappropriate antibiotic use.
